# Antipsychotic management in general practice: serial cross-sectional study (2011–2020)

**DOI:** 10.3399/BJGP.2024.0367

**Published:** 2024-11-26

**Authors:** Alan Woodall, Alex Gampel, Lauren E Walker, Frances S Mair, Sally Sheard, Pyers Symon, Iain Buchan

**Affiliations:** Department of Primary Care and Mental Health, Institute of Population Health, University of Liverpool, Liverpool; clinical lead for integrated care, Powys Teaching Health Board, Bronllys, Powys.; Powys Teaching Health Board, Bronllys, Powys.; Centre for Experimental Therapeutics, University of Liverpool, Liverpool.; General Practice and Primary Care, School of Health and Wellbeing, University of Glasgow, Glasgow.; Department of Public Health, Policy and Systems, Institute of Population Health, University of Liverpool, Liverpool.; National Institute for Health and Care Research (NIHR) Mental Health Research for Innovation Centre, University of Liverpool, Liverpool.; NIHR Mental Health Research for Innovation Centre, University of Liverpool, Liverpool; director, Civic Health Innovation Labs, University of Liverpool, Liverpool.

**Keywords:** antipsychotics, cardiometabolic risk factors, cross-sectional studies, general practice, medication review, psychiatry

## Abstract

**Background:**

Long-term use of antipsychotics confers increased risk of cardiometabolic disease. Ongoing need should be reviewed regularly by psychiatrists.

**Aim:**

To explore trends in antipsychotic management in general practice, and the proportions of patients prescribed antipsychotics receiving psychiatrist review.

**Design and setting:**

Serial cross-sectional study using linked general practice and hospital data in Wales (2011–2020).

**Method:**

Participants were adults (aged ≥18 years) registered with general practices in Wales. Outcome measures were prevalence of patients receiving ≥6 antipsychotic prescriptions annually, the proportion of patients prescribed antipsychotics receiving annual psychiatrist review, and the proportion of patients prescribed antipsychotics who were registered on the UK serious mental illness, depression, and/or dementia registers, or not on any of these registers.

**Results:**

Prevalence of adults prescribed long-term antipsychotics increased from 1.055% (95% confidence interval [CI] = 1.041 to 1.069) in 2011 to 1.448% (95% CI = 1.432 to 1.464) in 2020. The proportion receiving annual psychiatrist review decreased from 59.6% (95% CI = 58.9 to 60.4) in 2011 to 52.0% (95% CI = 51.4 to 52.7) in 2020. The proportion of overall antipsychotic use prescribed to patients on the serious mental illness register decreased from 50.0% (95% CI = 49.4 to 50.7) in 2011 to 43.6% (95% CI = 43.0 to 44.1) by 2020.

**Conclusion:**

Prevalence of long-term antipsychotic use is increasing. More patients are managed by GPs without psychiatrist review and are not on monitored disease registers; they thus may be less likely to undergo cardiometabolic monitoring and miss opportunities to optimise or deprescribe antipsychotics. These trends pose risks for patients and need to be addressed urgently.

## Introduction

Antipsychotics are licensed in the UK for the management of serious mental illness (SMI), such as schizophrenia and bipolar disorder.^1^ Some antipsychotics have UK licences for treatment of depression (quetiapine) and behavioural management in dementia (risperidone).^2^ Antipsychotics are also often prescribed, off-licence and long term, for people with personality disorder,^3^ behavioural management in individuals with learning disability,^4^ autistic people,^5^ and people with anxiety,^6^ in contravention of guidelines issued by the National Institute for Health and Care Excellence (NICE).^3^^,^^7^^,^^8^

Antipsychotics are effective medications for SMI, but long-term use increases risk of obesity,^9^ diabetes mellitus,^10^ and cardiovascular disease.^11^^,^^12^ Psychiatrist-led review of antipsychotics should occur regularly to prevent overuse; patients with SMI die 15–20 years prematurely, mainly because of cardiovascular disease and cancer,^13^^–^17 but their mortality is higher in many other conditions, such as severe COVID-19 infection.^18^ Avoidable early mortality for patients with SMI is of significant international public health concern;^19^ patients with SMI experience lower screening rates, less monitoring for cardiometabolic risk, and fewer interventions when cardiovascular disease occurs.^20^ Poorer physical health outcomes for patients with SMI remain problematic, even in countries with universal health care.^21^

GPs in the UK provide physical health reviews of patients with SMI.^13^^,^^22^ These are conducted via annual recall of patients with diagnoses that encode them on the Quality and Outcomes Framework (QOF) SMI (QOF SMI) register (termed a ‘mental health’ register, but it only includes patients with psychotic illnesses and/or prescribed lithium). The QOF is a performance management programme to establish registers of patients with chronic illnesses who require enhanced care, and forms part of the UK GP contract (now excluding Scotland) (see Supplementary Table S1).^23^ The QOF SMI annual review includes monitoring of alcohol consumption, smoking, body mass index, blood pressure, blood glucose, and lipids to detect patients at risk of developing cardiometabolic diseases.^13^ However, patients taking antipsychotics for dementia, depression, or conditions such as anxiety or personality disorder are not captured on the QOF SMI register and may not receive cardiometabolic monitoring; a cohort study of 47 724 patients in 2016 found <50% of patients prescribed first generation antipsychotics had an SMI diagnosis.^7^ GPs report they lack competencies to optimise antipsychotics (for example, alter dose, switch, or stop) without input from psychiatrists undertaking regular reviews.^22^^,^^24^^,^^25^ Patients who are commenced on antipsychotics by psychiatrists are frequently discharged to general practice; 31% of patients with SMI were managed solely by general practice in 2009,^26^ and may take antipsychotics long term without further psychiatric review. In this study, three main research questions were addressed:

- Has the prevalence of long-term antipsychotic use in general practice changed over the decade just before the COVID-19 pandemic?
- Has the proportion of patients prescribed long-term antipsychotics receiving psychiatrist review changed?
- Has the proportion of people prescribed antipsychotics that are included on the QOF SMI register changed?

[Table table7]

**Table table7:** How this fits in

Antipsychotics are effective treatments for serious mental illness but their use increases risk of cardiometabolic disease, therefore need should be reviewed regularly. There is increasing use of antipsychotics for non-psychotic illness by psychiatrists who then discharge patients to care by GPs, often for patients who are not captured on disease registers that automate recall for cardiometabolic risk monitoring. This study demonstrates a rising burden of long-term antipsychotic prescribing in general practice, with nearly half of all patients not under annual psychiatrist review, and only 43.56% (95% confidence interval = 43.01 to 44.10) of long-term antipsychotic prescriptions being issued to patients coded on the Quality and Outcomes Framework serious mental illness register by 2020. Many patients taking antipsychotics may thus be missed for cardiometabolic screening and review. Policy changes are required to ensure regular effective review to prevent increasing morbidity and early mortality for patients prescribed long-term antipsychotics.

## Method

### Study design

A serial cross-sectional population study was undertaken with reference to the ‘Strengthening the Reporting of Observational Studies in Epidemiology’ (STROBE) guidelines.^27^ This methodology has been used in international studies of antipsychotic prescribing.^28^^,^^29^

### Study population and setting

Individuals registered with GP practices providing data to the Secure Anonymised Information Linkage (SAIL) databank formed the population. Analysis focused on adult (aged ≥18 years) use, subdivided by subspeciality: general adult psychiatry (aged 18–64 years) and older adult psychiatry (aged ≥65 years). Patient age was calculated on 1 January each year.

In this study, psychiatrist review was defined as occurring when a record of psychiatrist outpatient clinic contact with a registered medical practitioner or advanced nurse practitioner (including general adult, older adult, child and adolescent, forensic or learning disability psychiatry, and psychotherapy subspecialties), or psychiatric hospital admission, occurred in the 12 months preceding each 1 January. If neither contact occurred, then the authors defined antipsychotic management as being solely provided by GPs.

Two health boards of the seven in Wales could not provide psychiatric outpatient clinic returns for the study period; therefore, analysis of the proportions of patients prescribed antipsychotics who had received psychiatrist review was limited to five health boards providing clinic returns for 2011–2020. These seven health boards all had similar prevalences of SMI, depression, and dementia from QOF returns to Welsh Government.^30^ The COVID-19 pandemic disrupted normal outpatient services; hence this study focused on psychiatrist review up to 1 January 2020.

### Data sources

The SAIL databank was the data source. This is an expanding data repository of 500 million anonymised and encrypted individual-level records from primary and secondary healthcare sources, and sociodemographic data relevant to health. This includes national datasets covering the whole of Wales (approximately 3 million population).^31^^–^35 Patients registered with general practices providing data to SAIL were examined using the Welsh Longitudinal General Practice (WLGP) dataset. As of 2023, 87% of Welsh general practices provide historical electronic health record data to SAIL for each patient, covering 83% of the population.^36^ Outpatient clinic attendance was determined from the Outpatient Database for Wales. Psychiatric hospital admission was ascertained using the Patient Episode Database for Wales. The SAIL databank has been validated for use in studies on mental illness.^15^

Diagnoses for inclusion in QOF SMI, depression, and dementia registers recorded in GP records and prescriptions for antipsychotics (with a UK licence for use during the study period as listed in the *British National Formulary*^2^) were extracted using five-digit Read codes (version 2) from the WLGP dataset (see repository: https://github.com/alanwoodall/AMP-Epidemiology). Discussion with two psychiatrists confirmed that two antipsychotics were rarely prescribed for psychiatric purposes and are mainly used for physical health: prochlorperazine (antiemetic) and levomepromazine (palliative care); these were excluded from analysis. Parenteral (‘depot’) antipsychotics or clozapine issued by GPs were also captured, but these are usually prescribed directly by psychiatrists.

### Analysis

The primary outcome measure was number of adults issued antipsychotic prescriptions on ≥6 different days in each calendar year. Analysis by gender, ethnicity, and Index of Multiple Deprivation quintile (2019 Welsh Index of Multiple Deprivation) was also undertaken. Sensitivity analysis of antipsychotic script events per annum was undertaken to examine patterns of use (see repository); the authors defined ≥6 script events in a 12-month period as long-term antipsychotic use conservatively, based on the frequency of psychiatric outpatient review (usually biannually) and on the definition of regular use in other studies.^37^ Further analysis was undertaken to examine antipsychotic use by age (18–64 years and ≥65 years), to mirror the UK division between general adult and old age psychiatry subspecialities. The prevalence of the six most common antipsychotics prescribed was also determined.

For the proportion of people on antipsychotics receiving psychiatrist review, a sensitivity analysis was undertaken, varying the period to capture review occurrence up to 5 years. The authors chose 12 months as the period to define antipsychotic review by psychiatry.

The number of patients who had a lifetime history of diagnostic codes in the QOF SMI, QOF dementia, and/or QOF depression registers was determined, along with the number of patients on each disease register prescribed long-term antipsychotics. These three psychiatric illness QOF registers cover diagnoses for almost all licensed uses of antipsychotics in the UK.^2^ The number of patients prescribed antipsychotics not on any of these three psychiatric QOF registers was also determined.

### Statistical analysis

Statistical analysis was undertaken using StatsDirect (version 4.0.1). The Clopper–Pearson method was used for confidence intervals (CIs) of binomial proportions.^38^ Differences between binomial proportions were evaluated using the Miettinen–Nurminen method.^39^ Results are presented as the main effect with a 95% CI. A 5% significance level was used for hypothesis tests.

### Patient and public involvement

Public advisors for research with the Mental Health Research for Innovation Centre, University of Liverpool, provided oversight of the study.

## Results

### Changes in prevalence of long-term antipsychotic prescribing

Prevalence of adults exposed to antipsychotics increased from 1.055% (*n* = 21 907/2 076 839; 95% CI = 1.041 to 1.069) to 1.448% (*n* = 31 946/2 206 635; 95% CI = 1.432 to 1.464; Table 1 and Supplementary Figure S1). This increase is exclusively in the 18–64 years age group: 0.921% (*n* = 14 781/1 605 559; 95% CI = 0.906 to 0.936) in 2011 increasing to 1.458% (*n* = 23 838/1 634 511; 95% CI = 1.444 to 1.477) in 2020. Of individual antipsychotics, quetiapine was the most prescribed in those aged ≥18 years (increasing from 0.306% (*n* = 6349/2 076 839; 95% CI = 0.298 to 0.313) in 2011 to 0.559% (*n* = 12 328/2 206 635; 95% CI = 0.549 to 0.569) in 2020. For those aged ≥65 years, antipsychotic prevalence fell from 1.512% (*n* = 7126/471 280; 95% CI = 1.477 to 1.547) in 2011 to 1.417% (*n* = 8108/572 124; 95% CI = 1.387 to 1.448) in 2020. Risperidone was the most prescribed antipsychotic in this age group, increasing from 0.165% (*n* = 779/471 280; 95% CI = 0.154 to 0.177) in 2011 to 0.364% (*n* = 2082/572 124; 95% CI = 0.348 to 0.380) in 2020.

**Table 1.** Antipsychotic prevalence changes between 2011 and 2020^a^

**Group**	**All antipsychotics**		**Quetiapine**		**Olanzapine**		**Risperidone**		**Amisulpride**		**Aripiprazole**		**Haloperidol**	

	**2011**	**2020**	**2011**	**2020**	**2011**	**2020**	**2011**	**2020**	**2011**	**2020**	**2011**	**2020**	**2011**	**2020**
**All adults (aged ≥18 years)**														
Counts, *r*/*n*	21 907/2 076 839	31 946/2 206 635	6349/2 076 839	12 328/2 206 635	5412/2 076 839	7520/2 206 635	3236/2 076 839	5020/2 206 635	1531/2 076 839	1190/2 206 635	1354/2 076 839	4263/2 206 635	991/2 076 839	825/2 206 635
Prevalence, % (95% CI)	1.055 (1.041 to 1.069)	1.448 (1.432 to 1.464)	0.306 (0.298 to 0.313)	0.559 (0.549 to 0.569)	0.261 (0.254 to 0.268)	0.341 (0.333 to 0.348)	0.156 (0.150 to 0.161)	0.227 (0.221 to 0.234)	0.074 (0.070 to 0.078)	0.054 (0.051 to 0.057)	0.065 (0.062 to 0.069)	0.193 (0.187 to 0.199)	0.048 (0.045 to 0.051)	0.037 (0.035 to 0.040)
Difference, % (95% CI)	+0.393 (+0.372 to +0.414)		+0.253 (+0.241 to +0.266)		+0.080 (+0.070 to +0.091)		+0.071 (+0.063 to +0.080)		−0.020 (−0.025 to −0.015)		+0.128 (+0.121 to +0.135)		−0.011(−0.014 to −0.006)	

**General adults (aged 18–64 years)**														
Counts, *r*/*n*	14 781/1 605 559	23 838/1 634 511	4290/1 605 559	10 453/1 634 511	4237/1 605 559	5509/1 634 511	2457/1 605 559	2938/1 634 511	800/1 605 559	809/1 634 511	1097/1 605 559	3398/1 634 511	535/1 605 559	534/1 634 511
Prevalence, % (95% CI)	0.921 (0.906 to 0.936)	1.458 (1.444 to 1.477)	0.267 (0.259 to 0.275)	0.640 (0.627 to 0.652)	0.264 (0.256 to 0.272)	0.337 (0.328 to 0.346)	0.153 (0.147 to 0.159)	0.180 (0.173 to 0.186)	0.050 (0.047 to 0.053)	0.049 (0.046 to 0.053)	0.068 (0.064 to 0.072)	0.208(0.197 to 0.211)	0.033 (0.031 to 0.036)	0.033 (0.030 to 0.036)
Difference, % (95% CI)	+0.537 (+0.514 to +0.561)		+0.373 (+0.358 to +0.387)		+0.073 (+0.061 to +0.085)		+0.027 (+0.018 to +0.036)		0.000 (−0.005 to +0.005)		+0.140(+0.131 to +0.148)		0.000 (−0.000 to +0.000)	

**Older adults (aged ≥65 years)**														
Counts, *r*/*n*	7126/471 280	8108/572 124	1914/471 280	1876/572 124	1175/471 280	2011/572 124	779/471 280	2082/572 124	730/471 280	381/572 124	267/471 280	875/572 124	456/471 280	291/572 124
Prevalence, % (95% CI)	1.512 (1.477 to 1.547)	1.417 (1.387 to 1.448)	0.406 (0.388 to 0.424)	0.328 (0.313 to 0.343)	0.249 (0.236 to 0.264)	0.351 (0.337 to 0.367)	0.165 (0.154 to 0.177)	0.364 (0.348 to 0.380)	0.155 (0.144 to 0.167)	0.067 (0.060 to 0.073)	0.057 (0.050 to 0.064)	0.153 (0.143 to 0.163)	0.097 (0.088 to 0.011)	0.051 (0.045 to 0.057)
Difference, % (95% CI)	−0.095 (−0.141 to −0.049)		−0.078 (−0.101 to −0.055)		+0.103 (+0.081 to +0.124)		+0.199 (+0.178 to +0.219)		−0.088 (−0.101 to −0.076)		+0.096 (+0.084 to +0.109)		−0.046 (−0.056 to −0.037)	

a *Analysis based on number of patients prescribed regular antipsychotics (*r*) as a proportion of the at-risk population in a calendar year (*n*).*

Table 2 shows prevalence of antipsychotics analysed by sex, deprivation quintile, and ethnicity. Females were more likely than males to be prescribed long-term antipsychotics (2020 female:male prevalence ratio 1.09; 95% CI = 1.07 to 1.12). There was increasing prevalence of antipsychotic use in the most deprived compared with least deprived quintiles (prevalence ratio in 2011 was 2.30, increasing to 2.58 in 2020). When analysed by ethnicity, White individuals had a higher prevalence of antipsychotic use than other ethnic groups, with Asian individuals having the lowest prevalence (2020 Asian:White prevalence ratio 0.37; 95% CI = 0.33 to 0.42).

**Table 2.** Prevalence of antipsychotic use in 2011 and 2020 by subgroup characteristic of sex, ethnicity, and quintile of 2019 WIMD score^a^

**Subgroup**	**2011**			**2020**			**2020–2011**

	**Counts, *r*/*n***	**Prevalence, % (95% CI)**	**Prevalence ratio (95% CI)**	**Counts, *r*/*n***	**Prevalence, % (95% CI)**	**Prevalence ratio (95% CI)**	**Prevalence difference, % (95% CI)**
**WIMD quintile**							
1 (most deprived)	6112/397 630	1.54 (1.50 to 1.58)	2.30 (2.20 to 2.40)	9156/421 284	2.17 (2.13 to 2.21)	2.58 (2.48 to 2.68)	0.64 (0.58 to 0.69)
2	5136/411 764	1.25 (1.22 to 1.28)	1.87 (1.78 to 1.95)	7033/419 569	1.68 (1.64 to 1.72)	1.99 (1.91 to 2.07)	0.43 (0.38 to 0.48)
3	3828/407 578	0.94 (0.91 to 0.97)	1.40 (1.33 to 1.46)	5069/410 416	1.24 (1.21 to 1.27)	1.46 (1.40 to 1.53)	0.30 (0.25 to 0.34)
4	3200/377 925	0.85 (0.82 to 0.88)	1.27 (1.21 to 1.33)	4284/390 523	1.10 (1.07 to 1.13)	1.30 (1.24 to 1.36)	0.25 (0.21 to 0.29)
5 (least deprived)	2843/425 334	0.67 (0.65 to 0.69)	1.00 (0.95 to 1.05)	3582/424 598	0.84 (0.81 to 0.87)	1.00 (0.96 to 1.05)	0.18 (0.14 to 0.21)
Missing	788/56 608	1.39 (1.30 to 1.49)	2.08 (1.93 to 2.25)	2822/140 245	2.01 (1.94 to 2.08)	2.39 (2.27 to 2.51)	0.62 (0.50 to 0.74)

**Gender^b^**							
Female	11 741/1 048 240	1.12 (1.10 to 1.14)	1.13 (1.10 to 1.16)	16 826/1 114 501	1.51 (1.49 to 1.53)	1.09 (1.07 to 1.12)	0.39 (0.36 to 0.42)
Male	10 166/1 028 599	0.99 (0.97 to 1.01)	1.00 (0.97 to 1.03)	15 120/1 092 134	1.38 (1.36 to 1.40)	1.00 (0.98 to 1.02)	0.40 (0.37 to 0.43)

**Ethnicity**							
Asian	142/29 749	0.48 (0.41 to 0.57)	0.33 (0.28 to 0.39)	273/37 339	0.73 (0.65 to 0.82)	0.37 (0.33 to 0.42)	0.25 (0.14 to 0.37)
Black	118/9854	1.20 (1.00 to 1.43)	0.83 (0.69 to 0.99)	185/12 876	1.44 (1.25 to 1.66)	0.73 (0.63 to 0.84)	0.24 (−0.06 to 0.54)
Mixed	65/6102	1.07 (0.84 to 1.36)	0.74 (0.58 to 0.94)	126/8712	1.45 (1.22 to 1.73)	0.73 (0.62 to 0.87)	0.38 (0.01 to 0.74)
Other	60/8652	0.69 (0.53 to 0.89)	0.48 (0.37 to 0.62)	133/13 635	0.98 (0.83 to 1.16)	0.50 (0.42 to 0.59)	0.28 (0.03 to 0.52)
White	17 309/1 200 276	1.44 (1.42 to 1.46)	1.00 (0.98 to 1.02)	24 862/1 261 529	1.97 (1.95 to 1.99)	1.00 (0.98 to 1.02)	0.53 (0.50 to 0.56)
Missing	4213/822 176	0.51 (0.49 to 0.53)	0.36 (0.34 to 0.37)	6367/872 544	0.73 (0.71 to 0.75)	0.37 (0.36 to 0.38)	0.22 (0.19 to 0.24)

a *Analysis based on number of patients prescribed regular antipsychotics (*r*) as a proportion of the at-risk population (*n*) in the calendar year.*

b *Secondary suppression was used for <10 missing gender codes by imputing the values equally between male and females to reduce risk of disclosure. WIMD = Welsh Index of Multiple Deprivation.*

Supplementary Table S2 shows the counts of patients receiving long-term antipsychotics listed in the *British National Formulary* in 2011 and 2020, and their licensed indications. The proportion of long-term antipsychotic exposure that is accounted for by ‘second generation’ or ‘atypical’ antipsychotics increased from 79.7% in 2011 to 91.7% in 2020. Approximately 94% of antipsychotic exposures in 2020 are accounted for by six individual antipsychotics: quetiapine (37.1%), olanzapine (22.6%), risperidone (15.3%), aripiprazole (12.9%), amisulpride (3.6%), and haloperidol (2.5%).

### Proportion of patients prescribed long-term antipsychotics receiving psychiatric specialist review

The percentage of adults aged ≥18 years taking long-term antipsychotics who received psychiatrist review in the 12 months preceding each year (2011–2020) fell from 59.6% (*n* = 9574/16 053; 95% CI = 58.9 to 60.4) in 2011 to 52.0% (*n* = 11 894/22 855; 95% CI = 51.4 to 52.7) in 2020 (Table 3 and Supplementary Figure S2). The proportion of those aged 18–64 years taking long-term antipsychotics that received psychiatrist review decreased from 65.7% (*n* = 7028/10 691; 95% CI = 64.8 to 66.6) in 2011 to 55.0% (*n* = 9358/17 013; 95% CI = 54.3 to 55.8) in 2020. The greatest decrease in psychiatrist review in this age group occurred in patients prescribed quetiapine (−17.0%; 95% CI = −19.0 to −15.0). For patients aged ≥65 years, less than half underwent psychiatric review, decreasing from 47.5% (*n* = 2546/5362; 95% CI = 46.1 to 48.8) in 2011 to 43.4% (*n* = 2536/5842; 95% CI = 42.1 to 44.7) in 2020. The greatest decrease was seen for those taking amisulpride (−18.2%; 95% CI = −25.1 to −11.2)

**Table 3.** Proportion of patients taking long-term antipsychotics who have had a specialist psychiatrist review in previous 12 months^a^

**Group**	**All antipsychotics**		**Quetiapine**		**Olanzapine**		**Risperidone**		**Amisulpride**		**Aripiprazole**		**Haloperidol**	

	**2011**	**2020**	**2011**	**2020**	**2011**	**2020**	**2011**	**2020**	**2011**	**2020**	**2011**	**2020**	**2011**	**2020**
**Adults (aged ≥18 years)**														
Patients reviewed by psychiatrist, *r*/*n*	9574/16 053	11 894/22 855	2978/4786	4539/8940	2362/3698	2736/5272	1428/2466	1750/3607	751/1146	504/919	787/974	2102/3191	390/813	311/593
Patients reviewed by psychiatrist, % (95% CI)	59.6 (58.9 to 60.4)	52.0 (51.4 to 52.7)	62.2 (60.8 to 63.6)	50.8 (49.7 to 51.8)	63.9 (62.3 to 65.4)	51.9 (50.6 to 53.2)	57.9 (56.0 to 59.8)	48.5 (46.9 to 50.2)	65.5 (62.7 to 68.2)	54.8 (51.6 to 58.0)	80.8 (78.2 to 83.2)	65.9 (64.2 to 67.5)	48.0 (44.6 to 51.4)	52.4 (48.3 to 56.5)
Difference, % (95% CI)	−7.6 (−8.6 to −6.6)		−11.4 (−13.2 to −9.7)		−12.0 (−14.0 to −9.9)		−9.4 (−12.0 to −6.8)		−10.7 (−14.9 to −6.5)		−14.9 (−17.8 to −11.9)		+4.5(−0.8 to +9.7)	

**General adults (aged 18–64 years)**														
Patients reviewed by psychiatrist, *r*/*n*	7028/10 691	9358/17 013	2315/3339	3945/7538	1901/2900	2104/3882	1124/1903	1110/2141	432/585	384/609	679/818	1775/2593	263/431	223/357
Patients reviewed by psychiatrist, % (95% CI)	65.7 (64.8 to 66.6)	55.0 (54.3 to 55.8)	69.3 (67.8 to 70.9)	52.3 (51.2 to 53.5)	65.6 (63.8 to 67.3)	54.2 (52.6 to 55.8)	59.1 (56.9 to 61.3)	51.8 (49.7 to 54.0)	73.8 (70.3 to 77.4)	63.1 (59.2 to 66.9)	83.0 (80.4 to 85.6)	68.5 (66.7 to 70.2)	61.0 (56.4 to 65.6)	62.5 (57.4 to 67.5)
Difference, % (95% CI)	−10.7 (−11.7 to −9.5)		−17.0 (−19.0 to −15.0)		−11.4 (−13.7 to −9.0)		−7.3 (−10.3 to −4.2)		−10.7 (−16.1 to −5.5)		−14.5 (−18.1 to −11.0)		+1.5 (−8.3 to +5.4)	

**Older adults (aged ≥65 years)**														
Patients reviewed by psychiatrist, *r*/*n*	2546/5362	2536/5842	663/1447	594/1402	461/798	632/1370	304/563	640/1466	319/561	120/310	108/156	327/598	127/382	88/236
Patients reviewed by psychiatrist, % (95% CI)	47.5 (46.1 to 48.8)	43.4 (42.1 to 44.7)	45.8 (43.3 to 48.4)	42.4 (39.8 to 45.0)	57.8 (54.3 to 61.2)	46.1 (43.5 to 48.8)	54.0 (49.9 to 58.1)	43.7 (41.1 to 46.2)	56.9 (52.8 to 61.0)	38.7 (33.3 to 44.1)	69.2 (62.0 to 76.5)	54.7 (50.7 to 58.7)	33.2 (28.5 to 38.2)	37.3 (31.1 to 43.5)
Difference, % (95% CI)	−4.1 (−5.9 to −2.2)		−3.4 (−7.1 to +2.0)		−11.7 (−16.0 to −7.3)		−10.3 (−15.2 to −5.5)		−18.2 (−25.1 to −11.2)		−14.5 (−23.3 to −5.8)		4.0 (−3.7 to 11.9)	

a *Analysis based on number of patients prescribed antipsychotics who had received psychiatrist review (*r*) as a proportion of the number of patients prescribed antipsychotics in that calendar year (*n*).*

A sensitivity analysis of psychiatrist review was conducted by varying the period of time allowed up to 5 years before 1 January 2020 (see Supplementary Figure S3). For patients aged 18–64 years prescribed long-term antipsychotics in 2020, 22.9% had no psychiatrist contact in the previous 5 years. For patients aged ≥65 years, 29.9% of those taking antipsychotics long term in 2020 had no psychiatrist contact in the previous 5 years.

### Proportions of patients on the psychiatric QOF registers prescribed long-term antipsychotics

In 2020, one in 63 (1.59%; *n* = 35 105/2 206 635; 95% CI = 1.57 to 1.61) adults aged ≥18 years had a lifetime diagnostic code for inclusion on the QOF SMI register (0.17%; 95% CI = 0.15 to 0.19); one in five adults (20.7%; *n* = 457 709/2 206 635; 95% CI = 20.7 to 20.8) had a code for inclusion on the QOF depression register (5.6%; 95% CI = 5.6 to 5.7); and one in 130 adults (0.77%; *n* = 16 902/2 206 635; 95% CI = 0.76 to 0.78) had a code for inclusion on the QOF dementia register (0.09%; 95% CI = 0.07 to 0.11) (Table 4). For adults without codes for inclusion on these three psychiatric illness QOF registers, the proportion fell from 83.7% (*n* = 1 739 328/2 076 839; 95% CI = 83.7 to 83.8) in 2011 to 78.1% (*n* = 1 722 532/2 206 635; 95% CI = 78.0 to 78.1) in 2020.

**Table 4.** QOF registration prevalence changes for people aged ≥18 years between 2011 and 2020^a^

**QOF registers**	**2011**	**2020**
**SMI**		
Counts, *r*/*n*	29 487/2 076 839	35 105/2 206 635
Prevalence, % (95% CI)	1.42 (1.40 to 1.44)	1.59 (1.57 to 1.61)
Difference, % (95% CI)	+0.17 (+0.15 to +0.19)	

**Depression**		
Counts, *r*/*n*	313 156/2 076 839	457 709/2 206 635
Prevalence, % (95% CI)	15.1 (15.0 to 15.1)	20.7 (20.7 to 20.8)
Difference, % (95% CI)	+5.6 (+5.6 to +5.7)	

**Dementia**		
Counts, *r*/*n*	14 028/2 076 839	16 902/2 206 635
Prevalence, % (95% CI)	0.68 (0.66 to 0.69)	0.77 (0.76 to 0.78)
Difference, % (95% CI)	+0.09 (+0.07 to +0.11)	

**None of the above**		
Counts, *r*/*n*	1 739 328/2 076 839	1 722 532/2 206 635
Prevalence, % (95% CI)	83.7 (83.7 to 83.8)	78.1 (78.0 to 78.1)
Difference, % (95% CI)	−5.7 (−5.8 to −5.6)	

a *Analysis is based on the number of patients (*r*), who have ever been on the relevant register, as a proportion of the at-risk population (*n*) in the calendar year. QOF = Quality and Outcomes Framework. SMI = serious mental illness.*

Table 5 shows the proportion of patients aged ≥18 years prescribed long-term antipsychotics in each QOF register (also see Supplementary Figure S4). The proportion of patients on the QOF SMI register prescribed antipsychotics increased to 39.74% (*n* = 13 915/35 015; 95% CI = 39.13 to 40.15) in 2020 (2.47; 95% CI = 1.72 to 3.22) since 2011, with olanzapine the most common exposure in 2020 (11.91%; *n* = 4180/35 105; 95% CI = 11.57 to 12.25). The proportion of patients on the QOF dementia register prescribed antipsychotics decreased from 16.71% (*n* = 2344/14 028; 95% CI = 16.10 to 17.34) in 2011 to 12.86% (*n* = 2174/16 902; 95% CI = 12.37 to 13.38) in 2020, with risperidone the most common exposure in 2020 (5.35%; *n* = 904/16 902; 95% CI = 5.02 to 5.70). The proportion of patients on the QOF depression register prescribed antipsychotics increased from 3.37% (*n* = 10 552/313 156; 95% CI = 3.31 to 3.43) in 2011 to 4.12% (*n* = 18 852/457 709; 95% CI = 4.06 to 4.18) in 2020, with quetiapine the most common exposure in 2020. The proportion of patients not on any of these three QOF psychiatric illness registers prescribed antipsychotics increased from 0.255% (*n* = 4441/1 739 328; 95% CI = 0.248 to 0.263) in 2011 to 0.357% (*n* = 6147/1 722 532; 95% CI = 0.348 to 0.366) in 2020, with quetiapine the most common exposure in 2020 (0.126%; *n* = 2170/1 722 532; 95% CI = 0.121 to 0.131).

**Table 5.** Changes in long-term antipsychotic use in adult patients (aged ≥18 years) with a lifetime code on the QOF SMI, depression, and/or dementia registers, and those who are not on any of these three psychiatric illness QOF registers, between 2011 and 2020^a^

**Proportion of patients on QOF registers prescribed antipsychotic**	**All antipsychotics**		**Quetiapine**		**Olanzapine**		**Risperidone**		**Amisulpride**		**Aripiprazole**		**Haloperidol**	

	**2011**	**2020**	**2011**	**2020**	**2011**	**2020**	**2011**	**2020**	**2011**	**2020**	**2011**	**2020**	**2011**	**2020**
**SMI**														
Counts, *r*/*n*	10 960/29 487	13 915/35 105	2614/29 487	4024/35 105	3498/29 487	4180/35 105	1680/29 487	1771/35 105	846/29 487	846/35 105	974/29 487	2549/35 105	423/29 487	448/35 105
Proportion, % (95% CI)	37.17 (36.62 to 37.72)	39.74 (39.13 to 40.15)	8.86 (8.54 to 9.19)	11.46 (11.13 to 11.80)	11.86 (11.49 to 12.23)	11.91 (11.57 to 12.25)	5.70 (5.43 to 5.96)	5.04 (4.82 to 5.28)	2.87 (2.68 to 3.06)	2.41 (2.25 to 2.58)	3.30 (3.10 to 3.51)	7.26 (6.99 to 7.54)	1.43 (1.30 to 1.57)	1.28 (1.16 to 1.40)
Difference, % (95% CI)	+2.47 (+1.72 to +3.22)		+2.60 (+2.13 to +3.06)		+0.04 (−0.46 to +0.54)		−0.82 (−1.17 to −0.48)		−0.46 (−0.71 to −0.21)		+3.96 (+3.62 to +4.30)		−0.16 (−0.34 to +0.02)	

**Depression**														
Counts, *r*/*n*	10 552/313 156	18 852/457 709	3546/313 156	8754/457 709	2773/313 156	4371/457 709	1336/313 156	2028/457 709	619/313 156	559/457 709	738/313 156	2529/457 709	307/313 156	329/457 709
Proportion, % (95% CI)	3.37 (3.31 to 3.43)	4.12 (4.06 to 4.18)	1.13 (1.10 to 1.17)	1.91 (1.87 to 1.95)	0.89 (0.85 to 0.92)	0.95(0.93 to 0.98)	0.43 (0.41 to 0.45)	0.44 (0.42 to 0.46)	0.20 (0.18 to 0.21)	0.12 (0.11 to 0.13)	0.24 (0.22 to 0.25)	0.55 (0.53 to 0.57)	0.10 (0.09 to 0.11)	0.07 (0.06 to 0.08)
Difference, % (95% CI)	+0.75 (+0.66 to +0.83)		+0.78 (+0.72 to +0.84)		+0.07(+0.03 to +0.11)		+0.01 (−0.01 to +0.05)		−0.08 (−0.09 to −0.06)		+0.31 (+0.29 to +0.35)		−0.03 (−0.04 to −0.01)	

**Dementia**														
Counts, *r*/*n*	2344/14 028	2174/16 902	951/14 028	430/16 902	165/14 028	326/16 902	210/14 028	904/16 902	304/14 028	100/16 902	70/14 028	209/16 902	161/14 028	72/16 902
Proportion, % (95% CI)	16.71 (16.10 to 17.34)	12.86 (12.37 to 13.38)	6.78 (6.36 to 7.20)	2.54 (2.32 to 2.79)	1.18 (1.01 to 1.37)	1.93 (1.73 to 2.15)	1.50 (1.30 to 1.70)	5.35 (5.02 to 5.70)	2.17 (1.94 to 2.42)	0.59 (0.48 to 0.71)	0.50 (0.40 to 0.63)	1.24 (1.07 to 1.40)	1.15 (0.98 to 1.34)	0.43 (0.33 to 0.52)
Difference, % (95% CI)	−3.85 (−4.64 to −3.06)		−4.24 (−4.70 to −3.77)		+0.75 (+0.47 to +1.03)		+3.85 (+3.43 to +4.27)		−1.58 (−1.83 to −1.32)		+0.74 (+0.53 to +0.95)		−0.72 (−0.92 to −0.53)	

**None of the above**														
Counts, *r*/*n*	4441/1 739 328	6147/1 722 532	1206/1 739 328	2170/1 722 532	750/1 739 328	1120/1 722 532	872/1 739 328	1585/1 722 532	245/1 739 328	149/1 722 532	144/1 739 328	611/1 722 532	315/1 739 328	205/1 722 532
Proportion, % (95% CI)	0.255 (0.248 to 0.263)	0.357 (0.348 to 0.366)	0.069 (0.065 to 0.073)	0.126 (0.0121 to 0.0131)	0.043 (0.040 to 0.046)	0.065 (0.061 to 0.069)	0.050 (0.047 to 0.054)	0.092 (0.088 to 0.097)	0.014 (0.012 to 0.016)	0.009 (0.007 to 0.010)	0.008 (0.007 to 0.010)	0.035 (0.033 to 0.038)	0.018 (0.016 to 0.020)	0.012 (0.010 to 0.014)
Difference, % (95% CI)	*+*0.102 (+0.090 to +0.113)		+0.057 (+0.051 to +0.063)		+0.022 (+0.017 to +0.027)		+0.042 (+0.036 to +0.048)		−0.005 (−0.008 to −0.003)		+0.027(+0.024 to +0.030)		−0.006 (−0.009 to −0.004)	

a *The number of patients with a lifetime code on the relevant QOF registers exposed to antipsychotics (*r*) is expressed as a proportion of the total population with a lifetime code for inclusion on each QOF register (*n*). QOF = Quality and Outcomes Framework. SMI = serious mental illness.*

### Proportion of overall long-term antipsychotic use by QOF register status

Table 6 shows the proportion of overall long-term antipsychotic use for patients who were registered on the QOF SMI, QOF dementia, and/or QOF depression registers, and the proportion of overall long-term antipsychotic use for patients not on any of these three QOF registers (also see Supplementary Figure S5). The proportion of overall antipsychotic use for patients on the QOF SMI register fell from 50.03% (*n* = 10 960/21 907; 95% CI = 49.37 to 50.69) in 2011 to 43.56% (*n* = 13 915/31 946; 95% CI = 43.01 to 44.10) in 2020. The proportion of overall antipsychotic use for patients on the QOF dementia register fell from 10.70% (*n* = 2344/21 907; 95% CI = 10.29 to 11.11) in 2011 to 6.81% (*n* = 2174/31 946; 95% CI = 6.53 to 7.09) in 2020. The proportion of antipsychotic use for patients on the QOF depression register increased from 48.17% (*n* = 10 552/21 907; 95% CI = 47.51 to 48.83) in 2011 to 59.01% (*n* = 18 852/31 946; 95% CI = 58.47 to 59.55) in 2020; 71.01% (*n* = 8754/12 328; 95% CI = 70.21 to 71.80) of total quetiapine use was for patients who had a lifetime history of being on the QOF depression register in 2020. The proportion of antipsychotics prescribed for patients not on these three registers decreased slightly from 20.27% (*n* = 4441/21 907; 95% CI = 19.74 to 20.81) in 2011 to 19.24% (*n* = 6147/31 946; 95% CI = 18.81 to 19.67) in 2020.

**Table 6.** Changes in proportion of overall antipsychotic use in adult patients (aged ≥18 years) with a lifetime code on the QOF SMI, depression, and/or dementia registers, and those who are not on any of these three psychiatric illness QOF registers, between 2011 and 2020^a^

**Proportion of total antipsychotic use by QOF register**	**All antipsychotics**		**Quetiapine**		**Olanzapine**		**Risperidone**		**Amisulpride**		**Aripiprazole**		**Haloperidol**	

	**2011**	**2020**	**2011**	**2020**	**2011**	**2020**	**2011**	**2020**	**2011**	**2020**	**2011**	**2020**	**2011**	**2020**
**SMI**														
Counts, *r*/*n*	10 960/21 907	13 915/31 946	2614/6349	4024/12 328	3498/5412	4180/7520	1680/3236	1771/5020	846/1530	846/1190	974/1354	2549/4263	423/911	448/825
Prevalence, % (95% CI)	50.03 (49.37 to 50.69)	43.56 (43.01 to 44.10)	41.17 (39.97 to 42.39)	32.64 (31.82 to 33.47)	64.63 (63.35 to 65.90)	55.59 (54.46 to 56.71)	51.92 (50.19 to 53.63)	35.28 (33.97 to 36.61)	55.29 (52.79 to 57.77)	71.09 (68.45 to 73.67)	71.94 (69.48 to 74.33)	59.79 (58.31 to 61.27)	46.43 (43.22 to 49.68)	54.30 (50.89 to 57.67)
Difference % (95% CI)	−6.47 (−7.33 to −5.61)		−8.53 (−9.98 to −7.08)		−9.04 (−10.77 to −7.33)		−16.64 (−18.82 to −14.46)		+15.80 (+12.12 to +19.47)		−12.15 (−15.10 to −9.18)		+7.87 (+3.16 to +12.58)	

**Depression**														
Counts, *r*/*n*	10 552/21 907	18 852/31 946	3546/6349	8754/12 328	2773/5412	4371/7520	1336/3236	2028/5020	619/1530	559/1190	738/1354	2529/4263	307/911	329/825
Prevalence, % (95% CI)	48.17 (47.51 to 48.83)	59.01 (58.47 to 59.55)	55.85 (54.63 to 57.07)	71.01 (70.21 to 71.80)	51.24 (49.91 to 52.57)	58.13(57.00 to 59.24)	41.29 (39.59 to 42.98)	40.40 (39.05 to 41.76)	40.46 (38.00 to 42.92)	46.97 (44.15 to 49.82	54.51 (51.84 to 57.16)	59.32 (57.85 to 60.80)	33.70 (30.70 to 36.83)	39.88(36.52 to 43.31)
Difference, % (95% CI)	+10.84 (+9.99 to +11.70)		+15.16 (+13.72 to +16.59)		+6.89(+5.15 to +8.62)		−0.89 (−3.06 to +1.28)		+6.51 (+2.76 to +10.27)		+4.81 (+1.80 to +7.84)		+6.18(+1.64 to +10.71)	

**Dementia**														
Counts, *r*/*n*	2344/21 907	2174/31 946	951/6349	430/12 328	165/5412	326/7520	210/3236	904/5020	304/1530	100/1190	70/1354	209/4263	161/911	72/825
Prevalence, % (95% CI)	10.70 (10.29 to 11.11)	6.81 (6.53 to 7.09)	14.98 (14.11 to 15.88)	3.49 (3.18 to 3.83)	3.05 (2.62 to 3.54)	4.34 (3.90 to 4.82)	6.49 (5.69 to 7.39)	18.01 (16.97 to 19.07)	19.87 (17.95 to 21.94)	8.40 (6.96 to 10.13)	5.17 (4.11 to 6.48)	4.90 (4.29 to 5.59)	17.67 (15.33 to 20.28)	8.73 (7.00 to 10.85)
Difference, % (95% CI)	−3.89 (−4.37 to −3.42)		−11.49 (−12.28 to −10.70)		+1.29 (+0.60 to +1.94)		+11.52 (+10.01 to +13.03)		−11.47 (−14.16 to −8.77)		−0.27 (−1.60 to +1.06)		−8.94 (−12.16 to −5.73)	

**None of the above**														
Counts, *r*/*n*	4441/21 907	6147/31 946	1206/6349	2170/12 328	750/5412	1120/7520	872/3236	1585/5020	245/1530	149/1190	144/1354	611/4263	315/911	205/825
Prevalence, % (95% CI)	20.27 (19.74 to 20.81)	19.24 (18.81 to 19.67)	19.00 (18.05 to 19.98)	17.60 (16.94 to 18.28)	13.86 (12.96 to 14.80)	14.89 (14.11 to 15.72)	26.95 (25.42 to 28.48)	31.57 (30.29 to 32.86)	16.01 (14.26 to 17.94)	12.52 (10.76 to 14.52)	10.64 (9.10 to 12.39)	14.33 (13.31 to 15.42)	34.58 (31.56 to 37.72)	24.85 (22.02 to 27.91)
Difference, % (95% CI)	−1.03 (−1.71 to −0.35)		−1.39(−2.58 to −0.23)		+1.03 (−0.19 to +2.27)		+4.62 (+2.61 to +6.65)		−3.49 (−6.16 to −0.83)		+3.69 (+1.61 to +5.78)		−9.73 (−14.05 to −5.41)	

a *The number of patients with a lifetime code on the relevant QOF registers exposed to antipsychotics (*r*) is expressed as a proportion of the total number of patients exposed to antipsychotics (*n*). QOF = Quality and Outcomes Framework. SMI = serious mental illness.*

## Discussion

### Summary

This study shows long-term antipsychotic prevalence increased in adults, with rises in those aged 18–64 years, and decreased in the aged ≥65 years population. Prevalence was higher in females, those from the most deprived quintile, and White ethnicity groups. For adults prescribed long-term antipsychotics, the proportion who had annual psychiatrist review decreased from 59.6% (95% CI = 58.9 to 60.4) in 2011 to 52.0% (95% CI = 51.4 to 52.7) in 2020; this decrease was more pronounced among older patients. The proportion of overall antipsychotic use prescribed to patients on the SMI register decreased from 50.0% (95% CI = 49.4 to 50.7) in 2011 to 43.6% (95% CI = 43.0 to 44.1) by 2020, meaning fewer patients would receive recall for cardiometabolic monitoring.

The current study suggests that antipsychotics are increasingly used for unlicensed and/or non-psychotic conditions. The decrease in psychiatrist reviews of patients prescribed antipsychotics, along with increasing use for conditions that are not on the QOF SMI register, may act synergistically to increase the risk of people becoming ‘trapped’ on antipsychotics in general practice and increasing their risk of cardiometabolic morbidity. This risk is worse for patients who are more deprived *—* antipsychotic prevalence was 2.58 (95% CI = 2.48 to 2.68) times higher in the most deprived compared with the least deprived quintile *—* and these inequalities have increased over the past decade. Given the prevalence of cardiometabolic disease is highest in the most deprived populations,^40^ this compounds the multimorbidity risk for this group.

### Strengths and limitations

To the best of the authors’ knowledge, this is the first study of long-term antipsychotic use to report the proportions of patients who are managed solely in general practice, the first to compare antipsychotic prescribing patterns between different QOF psychiatric illness registers, and of patients not on any psychiatric illness registers. Strengths include the focus on patients prescribed long-term antipsychotics who are most at risk of cardiometabolic disease; and the SAIL databank allows patient record linkage between general practice and secondary care datasets, so identifying management trends between general practice and psychiatric services.

Limitations include SAIL obtains data exclusively from Wales, so caution is needed extrapolating findings to other populations; SAIL cannot extract number of tablets issued per script, so accurate quantification of antipsychotic burden is not possible; SAIL only captures antipsychotic exposure when patients receive prescriptions from NHS general practice — it does not capture prescriptions issued from private services, for patients registered with prison or military general practices, and when scripts are issued by secondary care, as is the case for most parenteral antipsychotic prescriptions. Additionally, clozapine is licensed in the UK only for issue by psychiatrists so would not be routinely captured. Only eight patients received long-term clozapine scripts from general practice in 2020 (see Supplementary Table S2), but the clozapine monitoring service for Wales had 1984 patients registered in 2019;^41^ these represent missing data of antipsychotic exposure that the study was not able to capture. The study has not identified patients prescribed ≥2 antipsychotics concurrently.

For psychiatrist review, the authors cannot identify ‘indirect’ management between GPs and psychiatrists (for example, communication without patient contact), nor will it have identified patient contact with non-prescribing mental health professionals, who may provide indirect input into antipsychotic review. Although the study used lifetime prevalence of diagnostic codes to determine patient exposure to QOF SMI, QOF dementia, and QOF depression registers, as other studies have done,^7^ this will not detect changes in psychiatric diagnoses that can happen over time. Further, psychiatric codes may be inaccurate.^42^ Erroneous diagnoses may not be removed from GP records when a subsequent correct one is added.^43^ Incomplete, inaccurate, or non-contemporaneous coding of psychiatric conditions to measure effectiveness of registers to improve health outcomes is a concern of many UK and international studies, and must be considered when drawing conclusions from data.^44^^–^47 Finally, limited ethnicity coding capture for the population within SAIL databank (61% had ethnicity coding in 2020 using primary and secondary care datasets) reduced the authors’ ability to draw conclusions for this subgroup.

However, the tentative trends reported in the present study of similar or higher rates of antipsychotic use in White people compared with other ethnic groups have been previously reported.^48^ Studies confirm Wales has relatively poor primary care coding of ethnicity (40%) compared with England (75%), and work is underway to improve ethnicity recording in SAIL to nearly 95%.^49^

### Comparison with existing literature

The study found an increased prevalence of adults prescribed long-term antipsychotics. The prevalences are consistent with other studies; Marston *et al* reported an incidence of 0.7% exposure to antipsychotics in their cohort study, which ended in 2011;^7^ Shoham *et al* reported antipsychotic prevalences of 1.2% in 2014, from a questionnaire survey of patients aged ≥16 years.^50^ The greatest increase in use was seen for quetiapine, which accounted for 43.9% (*n* = 10 453/23 838; 95% CI = 43.2 to 44.5) of all antipsychotic exposures in the 18–64 years age group by 2020. This mirrors findings from international cross-sectional studies: Wilkinson and Mulder found overall antipsychotic prevalence had risen from 1.88% to 2.81% between 2008 and 2015 in New Zealand;^28^ Hálfdánarson *et al* reported increasing overall antipsychotic prevalence in 16 countries’ ranging from 0.32% in Columbia to 7.8% in Taiwan by 2014;^29^ these studies report antipsychotic prescribing from primary and secondary care. Despite this increase in antipsychotic use, psychiatric illness QOF register prevalences have not increased proportionately; this suggests increasing antipsychotic use for non-psychotic disorders, such as anxiety, depression, and personality disorder, as has been reported.^3^^,^^7^^,^^43^ Limited access to psychological therapies may be increasing antipsychotic use for non-psychotic disorders.^22^

Prevalence of long-term antipsychotic exposure in patients aged ≥65 years has decreased slightly, with significant falls in patients with dementia (−3.85%; 95% CI = −4.64 to −3.06); this is promising, given the increased risk of mortality when antipsychotics are used in Alzheimer’s dementia.^51^ However, long-term risperidone prevalence in dementia has increased (+3.85%; 95% CI = +3.43 to +4.27). The UK licence for risperidone is for *‘Short-term treatment (up to 6 weeks) of persistent aggression in patients with moderate to severe Alzheimer’s dementia’*,^2^ and yet one in 19 patients with dementia were prescribed risperidone long term; a study by Woodall *et al* reports pressure on clinicians to use antipsychotics for patients with dementia in care homes.^22^ Recent studies suggest this trend of decreasing overall antipsychotic use in patients with dementia was not maintained during the COVID-19 pandemic,^52^ and international studies have not all reported this decrease.^28^^,^^29^

The current study found decreasing proportions of patients prescribed antipsychotics have an annual psychiatrist review. One explanation may be more antipsychotics are being prescribed by GPs independently; in the current study it was not possible to determine which clinicians initiated antipsychotics, but qualitative studies suggest GPs lack confidence to initiate and manage antipsychotics without psychiatrist input,^22^^,^^25^^,^^53^ so the authors of the current study suspect this is unlikely. Another possible explanation is that more patients are discharged on antipsychotics by psychiatrists to general practice, either when patients with SMI are ‘stable’ from a psychiatric perspective, or when antipsychotics are prescribed to patients who do not have psychotic illness.

Although the prevalence of antipsychotic exposure is increasing, the proportion of total antipsychotic use for patients with a history of SMI is decreasing; this study found that less than half of antipsychotic use (43.56% in 2020) was for patients with a history of SMI, similar to the proportions used for SMI reported by Marston *et al.*^7^ This supports the authors of the current study’s concern that more antipsychotics are being used long term for non-psychotic disorders in contravention of NICE guidelines (for example, for personality disorder, where antipsychotics should only be used short term^54^). The data show the greatest increase in antipsychotic prescribing was for patients with a history of depression; whether antipsychotics are being used solely to treat depressive disorders, or the patient has a comorbid SMI or dementia code, cannot be determined. Cohort studies are needed to examine antipsychotic use for patients with exclusive depressive diagnoses to determine trends.

Only the QOF SMI register funds GPs to undertake cardiometabolic monitoring of patients taking antipsychotics; the majority of patients prescribed long-term antipsychotics are not on this register, and GPs are not funded to undertake cardiometabolic monitoring for them. The authors suspect many GPs still perform unfunded cardiometabolic monitoring when antipsychotics are used in non-SMI conditions. However, with most cardiometabolic monitoring devolved to GP practices, even when a patient remains under psychiatrist care, there is a risk that patients taking antipsychotics long term may not have cardiometabolic monitoring undertaken, as general practice utilises automated QOF SMI register recalls. Patients on the QOF SMI register will get annual recalls for cardiometabolic monitoring and GP review. For patients on the QOF dementia or QOF depression registers taking antipsychotics, there is no QOF requirement to undertake cardiometabolic monitoring, so this is less likely to happen. For patients prescribed long-term antipsychotics not captured on any QOF psychiatric illness register (for example, personality disorder, anxiety, or autism), there is no funding or automated recall for GP review or cardiometabolic monitoring. GPs are supposed to conduct annual medication reviews, but research shows that these are often of variable effectiveness, particularly when antipsychotics are prescribed and there is no ongoing psychiatric input.^22^^,^^25^^,^^53^^,^^55^ A recent study in Scotland showed specialist pharmacist-led physical health monitoring of patients prescribed antipsychotics can improve cardiometabolic monitoring and proactive intervention to ameliorate risk factors.^56^

### Implications for research and practice

This study demonstrated a rising burden of antipsychotic use in UK general practice. Further research is required to determine antipsychotic initiation rates by GPs as opposed to psychiatrists, and to determine antipsychotic prevalence in different ethnic groups, and specific use of antipsychotics for non-psychotic conditions such as anxiety, depression, autism, and learning disability.^22^^,^^25^^,^^53^ As healthcare services recover from the COVID-19 pandemic, future studies will need to determine if these concerning trends in antipsychotic management continue to worsen, to further inform future health and social care policy.

However, this current study has shown worrying findings: fewer patients are receiving annual psychiatric review, as more are discharged to general practice for sole management. With the usual cautions around extrapolation, the authors estimate that in 2020, based on a UK midpoint adult population estimate of 52 million,^57^ an antipsychotic prevalence of between 1.4%–1.5%, and about half of those patients cared for solely by GPs, approximately 360 000 to 390 000 patients may be being prescribed antipsychotics long term without regular psychiatric review. Furthermore, antipsychotics are being prescribed for non-psychotic illnesses that are not captured on the QOF SMI register, meaning cardiometabolic monitoring may not occur. Therefore, there is an urgent need for targeting of resources to enable patients taking antipsychotics long term to have access to an annual review with a psychiatrist able to optimise or withdraw antipsychotics. Furthermore, general practice funding for cardiometabolic monitoring should be based on antipsychotic use rather than diagnosis. Changes in policy are needed to prevent patients being ‘trapped’ on antipsychotics long term without psychiatrist review and ensure adequate cardiometabolic monitoring if the premature mortality experienced by patients with psychiatric illnesses is to be addressed.

## Data

Access to the underlying potentially identifiable pseudo-anonymised electronic health records datasets held by SAIL databank is restricted by best practice, and requests for access to the datasets must be submitted to the SAIL databank information governance team, information for which can be found at: https://saildatabank.com/governance/approvals-public-engagement/information-governance. All of the supplementary data, coding examples, and code lists are available at the following Github repository: https://github.com/alanwoodall/AMP-Epidemiology.
